# Long-latency TMS-evoked potentials during motor execution and inhibition

**DOI:** 10.3389/fnhum.2013.00751

**Published:** 2013-11-12

**Authors:** Kentaro Yamanaka, Hiroshi Kadota, Daichi Nozaki

**Affiliations:** ^1^Graduate School of Human Life Sciences, Showa Women’s UniversityTokyo, Japan; ^2^Graduate School of Education, The University of TokyoTokyo, Japan; ^3^Research Institute, Kochi University of TechnologyKochi, Japan

**Keywords:** transcranial magnetic stimulation, electroencephalography, motor-evoked potentials, motor cortex, execution, inhibition

## Abstract

Transcranial magnetic stimulation (TMS) has often been used in conjunction with electroencephalography (EEG), which is effective for the direct demonstration of cortical reactivity and corticocortical connectivity during cognitive tasks through the spatio-temporal pattern of long-latency TMS-evoked potentials (TEPs). However, it remains unclear what pattern is associated with the inhibition of a planned motor response. Therefore, we performed TMS-EEG recording during a go/stop task, in which participants were instructed to click a computer mouse with a right index finger when an indicator that was moving with a constant velocity reached a target (go trial) or to avoid the click when the indicator randomly stopped just before it reached the target (stop trial). Single-pulse TMS to the left (contralateral) or right (ipsilateral) motor cortex was applied 500 ms before or just at the target time. TEPs related to motor execution and inhibition were obtained by subtractions between averaged EEG waveforms with and without TMS. As a result, in TEPs induced by both contralateral and ipsilateral TMS, small oscillations were followed by a prominent negative deflection around the TMS site peaking at approximately 100 ms post-TMS (N100), and a less pronounced later positive component (LPC) over the broad areas that was centered at the midline-central site in both go and stop trials. However, compared to the pattern in go and stop trials with TMS at 500 ms before the target time, N100 and LPC were differently modulated in the go and stop trials with TMS just at the target time. The amplitudes of both N100 and LPC decreased in go trials, while the amplitude of LPC decreased and the latency of LPC was delayed in both go and stop trials. These results suggested that TMS-induced neuronal reactions in the motor cortex and subsequent their propagation to surrounding cortical areas might change functionally according to task demand when executing and inhibiting a motor response.

## INTRODUCTION

Transcranial magnetic stimulation (TMS; Barker et al., 1985) is a powerful tool that allows for the non-invasive investigation of the functional state of the cerebral cortex and corticomotoneuronal (CM) pathways (Hallett, 2000, 2007; Walsh and Cowey, 2000; Reis et al., 2008). Motor evoked potentials (MEP) that are induced in hand muscles after TMS over the motor cortex can be modulated during various motor tasks. For example, pre-movement MEP enhancements within 100 ms before response onset have been reported in many previous studies (Starr et al., 1988; Pascual-Leone et al., 1992; Chen et al., 1998; Leocani et al., 2000; Yamanaka et al., 2002). In contrast, the transient suppression of MEPs has been demonstrated during no-go trials of go/no-go tasks (Hoshiyama et al., 1996, 1997; Leocani et al., 2000; Yamanaka et al., 2002) and during the stop trials of stop-signal tasks (Badry et al., 2009; van den Wildenberg et al., 2010). The results of those studies have suggested that such MEP changes might primarily reflect modulations of CM excitability according to task demand.

Transcranial magnetic stimulation has often been used in combination with electroencephalographic (EEG) recordings (Ilmoniemi et al., 1997). This combined TMS-EEG technique makes it possible to investigate cortical reactivity and corticocortical connectivity from the spatiotemporal patterns of TMS-evoked potentials (TEP), which consist of peaks of negative/positive oscillations lasting about 300 ms (Komssi and Kähkönen, 2006; Ilmoniemi and Kičić, 2010). Although the functional meaning and cortical origin of the TEP peaks are not completely understood, a prominent long-latency negative peak has been commonly observed when TMS is delivered over the motor cortex in many previous studies (Paus et al., 2001; Nikulin et al., 2003; Komssi et al., 2004; Bonato et al., 2006; Kičić et al., 2008; Bonnard et al., 2009; Lioumis et al., 2009; Ferreri et al., 2011, 2012; Rogasch et al., 2013). This reproducible large negative peak at about 100 ms after the TMS pulse is named N1 or N100.

Previous studies have demonstrated that TEPs are modulated in various conditions, including arousal states (Massimini et al., 2005, 2007) and during the performance of motor tasks (Nikulin et al., 2003; Kičić et al., 2008;; Bonnard et al., 2009). Nikulin et al. (2003) have reported that the N100 peak that is induced by TMS over the motor cortex that is contralateral to the response hand is attenuated during a visually triggered motor response task. Kičić et al. (2008) have demonstrated that such N100 attenuation is observed during visually triggered motor response tasks with not only contralateral, but also ipsilateral, hand responses, although it is smaller in the ipsilateral hand response. These studies have commonly indicated that, during motor preparation and/or execution periods, MEP amplitudes increase, but the N100 amplitudes in TEPs to the motor cortex decrease. That is, the N100 of the TEPs to the motor cortex might be associated with cortical inhibitory processes (Nikulin et al., 2003; Bender et al., 2005; Kähkönen and Wilenius, 2007; Kičić et al., 2008). However, it is still unknown how the N100 in TEPs is modulated when inhibiting a planned motor response.

Human neuroimaging studies have reported that a scattering of cortical regions, comprised mesial, medial, and inferior frontal and parietal cortices, as well as motor cortex, were activated during tasks with motor inhibition (Garavan et al., 1999; Liddle et al., 2001; Rubia et al., 2001; Watanabe et al., 2002; Wager et al., 2005). However, the detailed time course of the motor inhibitory activities cannot be revealed by neuroimaging studies mainly due to the limitations of temporal resolution. Moreover, direct relationships between the motor cortex and motor inhibitory regions cannot be revealed by them. On the other hand, TMS-EEG study can be used for assessing cortical reactivity and corticocortical connectivity at the time when TMS is delivered.

Therefore, we conducted TMS-EEG recordings during a timing-coincident go/stop task (Coxon et al., 2006, 2007) and examined the differences in TEPs at the time of motor execution and inhibition. We especially focused on the N100 and the subsequent late positive component (LPC) in this study.

## MATERIALS AND METHODS

### PARTICIPANTS

Six right-handed healthy volunteers (six men, 27.9 ± 5.7 years) participated in contralateral-TMS session (over the left hemisphere). Another six right-handed healthy volunteers (one woman and five men, 26.9 ± 4.7 years) participated in the ipsilateral-TMS session (over the right hemisphere). All participants provided their informed consent, and the experimental procedures were approved by the local ethics committee of the Graduate School of Education at the University of Tokyo.

### TASK SETTING

**FIGURE 1:**
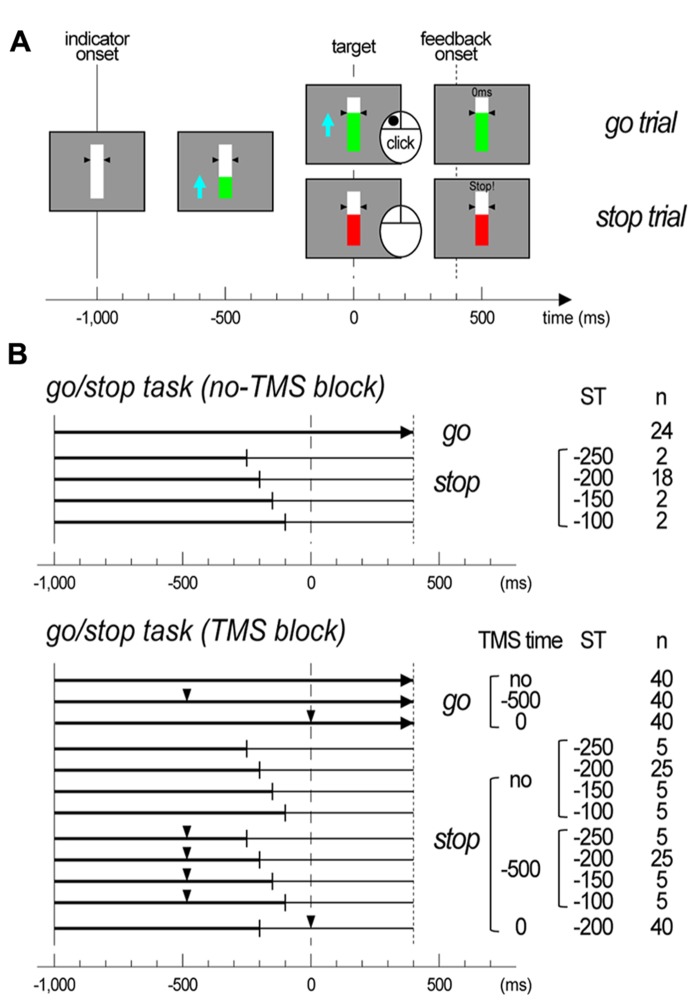
**Task designs of experiments. (A)** Illustrations of the display for the go/stop task. The trial type is noted on the right side, and the time scale is displayed at the bottom. **(B)** Illustrations of trial structure for the go/stop task with and without transcranial magnetic stimulation (TMS). The time scale is displayed at the bottom. The vertical lines at -1,000 ms represent the indicator onset, the vertical dashed lines at 0 ms represent the target, and the vertical dotted lines represent the feedback onset. The small triangles represent the time points at which TMS was delivered (TMS time). The trial type is noted on the right side, with the TMS time, stop time (ST), and total number of trials. In the stop trials, the time points at which an indicator stopped are shown with vertical thin bars.

All participants conducted a timing-coincident go/stop task (**Figure 1A**). In the task, each trial began with presentation of a white bar against a gray background with two small black triangles indicating a target at the center of the display. After 600 ms, a green indicator moved upward from the bottom of the bar at a constant rate, reaching the target (black triangles) in 1,000 ms and the top of the bar in 1,400 ms. The time point at which the indicator began moving upward was referred to as the indicator onset. Participants were instructed to click the mouse in order to stop the moving green indicator at the target (referred to as go trials). In half of the trials, the moving green indicator unexpectedly stopped and turned red just before it reached the target. The participant was instructed to withhold their click when the moving green indicator stopped and turned red (referred to as stop trials). The time point at which the indicator stopped (stop time: ST) was set at -250, -200, -150, and -100 ms relative to the target. In each go and stop trial, after 1,400 ms of the indicator onset, visual feedback about a participant’s performance [response time (RT) relative to target (ms) or “miss” for go trials; “stop!!” or “false alarm” with RT (ms) for stop trials] was presented for 500 ms on the central bar. This constant time setting was used to prevent participants’ eye blinks before the visual feedback onset. The participant was informed that the indicator in some trials would be easy to stop, and that it would be more difficult or impossible to stop in other trials because it would be too close to the target.

### TRANSCRANIAL MAGNETIC STIMULATION

Transcranial magnetic stimulation was applied over the left or right motor cortex with a Magstim 200 and a figure-8-shaped coil (Magstim Co. Ltd., Whitland, UK; maximum output, 1.5 T; 7 cm diameters). In order to keep the coil at the same position and direction against the scalp of a participant throughout the experiment, we used a mechanical arm and an elastic band. The coil over the left or right motor cortex was placed in the optimal position and direction in order to elicit MEPs in the right or left first dorsal interosseous (FDI) muscle. TMS intensity was expressed as a percentage of the motor threshold [MT; ±% of the maximal stimulator output: mean ± standard deviation (SD) for all participants], which was defined as the minimum intensity necessary to induce MEPs over 50 μV in the resting FDI muscle in at least three of five trials. TMS intensity in the experiment was set to the suprathreshold (120% of MT) in order to obtain MEPs of matched amplitudes (approximately 1.0 mV) in the resting FDI muscle. Coil position and MT were repeatedly checked and maintained throughout the experiment. All participants wore earplugs during the entire experiment to reduce the auditory click produced by the TMS coil.

### EEG AND EMG RECORDINGS

During the performance of the go/stop task, EEG, and electromyograms (EMG) were continuously acquired with a TMS-compatible EEG recording system (BrainAmp, Brain Products GmbH, Munich, Germany). EEG was recorded from 61 Ag/AgCl surface electrodes that were mounted on an elastic cap and that corresponded to the modified International 10–20 System of electrode placement, and four additional electrodes were attached to the forehead, left, and right ears, and the site beneath the left eye. In order to reduce the TMS-induced artifacts, we used the electrode lead wire rearrangement technique (Sekiguchi et al., 2011). The data were recorded against a reference electrode that was placed on the forehead and later re-referenced offline to the averaged value of the earlobes. The data from the site beneath the left eye was used for monitoring eye blink and eye movement. EMG was recorded from the right or left FDI muscle with Ag/AgCl surface electrodes. Electrode impedance was maintained below 10 kømega. EEG and EMG signals were amplified and filtered (bandpass settings: 0.5–100 Hz for EEG signals and 50–300 Hz for EMG signals) and continuously stored with a trigger signal from the computer that indicated task onset and TMS trigger at a sampling rate of 1,000 Hz for the offline analysis. In the offline analysis, we resampled all data at a rate of 500 Hz.

### EXPERIMENTAL PROCEDURES

Participants were comfortably seated on a chair in an electrically shielded room facing a 12.1-in. computer display (screen resolution, 1,280 × 800 pixels; refresh rate, 60 Hz), and she/he placed her/his right index finger on the main (left) button of a computer mouse. Then, she/he was lectured about the go/stop task, and she/he practiced it. After completing the experimental settings for the EEG and EMG recordings and TMS, participants first conducted 1 block of the go/stop task without TMS. This no-TMS block was conducted in order to assess task performance and record electrophysiological signals during the go/stop task without TMS. There were 48 trials in total, 24 go and 24 stop. The intertrial intervals were 4.5 s. For the stop trials, the indicator stopped randomly at an ST of -250, -200, -150, and -100 ms with 2, 2, 18, and 2 trials for each ST. Next, the participants conducted five blocks of the go/stop task with TMS. There were 240 trials in total, 120 go, and 120 stop. The intertrial intervals were 7.5 s, and the interblock intervals were about 3 min. In both the go and stop trials, TMS was randomly delivered at a TMS time of -500 and 0 ms relative to the target, with a total of 80 trials, 40 go and 40 stop, for each TMS time. That is, TMS was not delivered in 40 go and 40 stop trials in these five TMS blocks. We adopted a TMS time of -500 ms as a motor preparatory period for participants’ brain processes not to separate into go and stop, while a TMS time of 0 ms was adopted as the period for motor execution or inhibition. For the stop trials, the indicator stopped consistently at an ST of -200 ms in the stop trials with TMS at a TMS time of 0 ms or randomly at an ST of -250, -200, -150, and -100 ms, with 5, 5, 25, and 5 trials for each ST, in the stop trials with TMS at a TMS time of -500 ms or without TMS. Although the ratio of each ST that appeared in the stop trials in five TMS blocks was different at each TMS time, it was totally equal to those in a no-TMS block (8.3, 8.3, 75, and 8.3% for each ST). This biased setting in ST and TMS time was used to increase the number of stop trials with TMS at a TMS time of 0 m and at an ST of -200 ms for the EEG averaging procedure. None of the participants noticed this biased ST setting during the experiments. The numbers of trials in each ST and TMS time condition are shown in **Figure 1B**.

### PERFORMANCE DATA

Because the task performance in the trials with TMS was changed by the effects of TMS (an appearance of MEP and a silent period), we used only trials of the go/stop task without TMS for task performance assessments. In order to exclude premature responses and misses in the go trials, outlying RTs were discarded with the following criteria: <-100 and >150 ms in the go trials (0.7% for six subjects in the contralateral-TMS experiment and 0.5% for six subjects in the ipsilateral-TMS experiment). The means and SDs of the RTs were then calculated for each participant and trial condition. For the stop trials, the percentage of correct responses (% correct) was calculated for each participant and ST. Next, the ST for which the probability of successful stopping was 50% (50% ST) was determined with the least-square fitting curve to the sigmoid function. The 50% ST was subtracted from the mean go RT in order to determine the stop-signal reaction time (SSRT), which is the estimated time required for unobservable stop processes based on a *race model* (Logan and Cowan, 1984; Logan et al., 1984; Verbruggen and Logan, 2008).

### MEP DATA

For each trial with TMS, the peak-to-peak MEP amplitudes were measured from the EMG data. Stop trials with false alarm responses were excluded from the MEP analysis. The mean MEP amplitudes were calculated for each participant, trial condition (go or stop), and TMS time (-500 or 0 ms). Finally, the group mean MEP amplitudes were calculated for each trial condition and TMS time.

### EEG DATA PROCESSING

**Figure A1:**
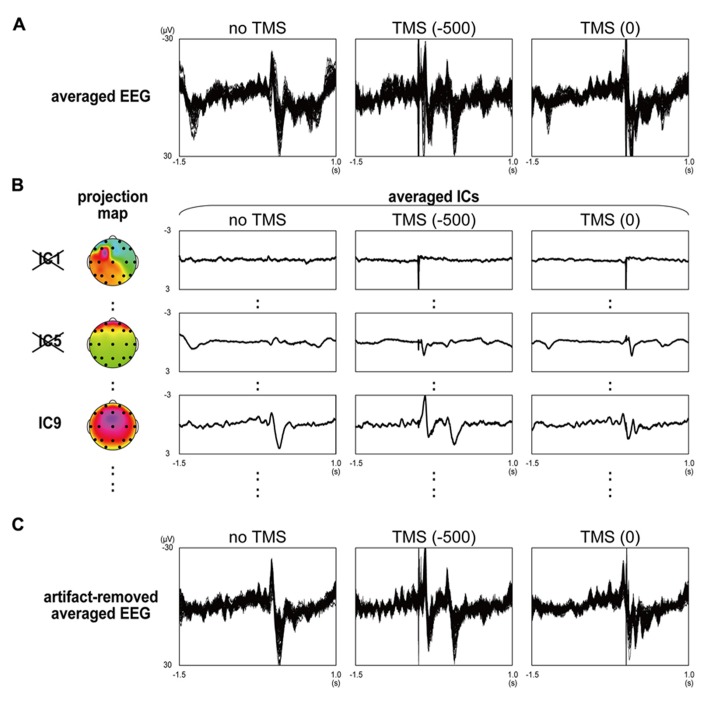
**Artifact rejection from TMS-EEG data by using independent component analysis in a typical participant. (A)** Averaged EEG waveforms in no-TMS stop trials (left panel), stop trials with contralateral-TMS at -500 and 0 ms (middle and right panels) recorded from 61 surface electrodes. **(B)** Averaged independent component (IC) waveforms and their projection maps in no-TMS stop trials (left panel), stop trials with contralateral-TMS at -500 and 0 ms (middle and right panels) extracted from 61 EEG waveforms. In order of their variance size, the largest component (IC1), fifth largest component (IC5), and ninth largest component (IC9) were selectively shown. **(C)** Artifact-removed averaged EEG waveforms in no-TMS stop trials (left panel), stop trials with contralateral-TMS at -500 and 0 ms (middle and right panels).

The data from the 61-channel scalp EEG (and 1-channel eye-related potential) in all conditions were first segmented in epochs from 1,500 ms before and 1,000 ms after the target time, and all of the segmented data was bunched together for each subject. Next, an independent component (IC) analysis with extended infomax algorithm (Bell and Sejnowski, 1995; Lee et al., 1999) was applied to the EEG data in order to identify and remove the components reflecting TMS-related artifacts and eye-blink- and/or eye-movement-related activities (Jung et al., 2000a,b; Johnson et al., 2012). From the 62 extracted independent components (ICs), the TMS-related ICs were chosen mainly by their time courses; the variance value of the IC during a time period of 20 ms just after the TMS was 20 times larger than those during the rest of the time periods and during no-TMS trials. The results suggested that such ICs impulsively induced huge potentials only when the TMS pulse was delivered. Eye-blink- and eye-movement-related activities were also determined by the time courses, which indicated their inactivation during a task, and scalp topographies of the projection maps, which provided their origin on the edge of anterior sites. Based on these criteria, we could effectively remove TMS-related [11.4 ± 2.7 (mean ± SD for all participants)], eye-blink-related (1 ± 0), and eye-movement-related (0.8 ± 0.4) components and obtain EEG waveforms with little distortion, at least during the time period with two large long-latency TEP components (see **Figure A1** in Appendix).

The artifact-removed EEG data was low-pass filtered below 40 Hz. Next, after the baseline correction (during the 500 ms before indicator onset), they were separately averaged for 2 trial (go/stop) × 3 TMS time (no-TMS/TMS at -500 ms/TMS at 0 ms) × 2 TMS side (contralateral/ipsilateral) conditions for each participant. For no-TMS stop trials, EEG data were averaged only in stop trials with an ST of -200 ms because the number of trials with STs of -250, -150, and -100 ms was so small for the averaging procedure. However, for stop trials with a TMS at -500 ms, the EEG data were collectively averaged in all stop trials with STs of -250, -200, -150, and -100 ms because the averaged EEGs around the TMS time (-500 ms) were little affected by the differences in the STs. Finally, in order to extract the TEP during the performance of the go/stop task, the averaged EEG waveforms in no-TMS go or stop trials were subtracted from those in go or stop trials with TMS at -500 or 0 ms (Nikulin et al., 2003; Kičić et al., 2008). For the figure representations, we obtained 61-channel grand-mean-averaged EEGs in no-TMS trials for 2 trial × 2 TMS side conditions and 61-channel grand-mean TEPs for 2 trial × 2 TMS time × 2 TMS side conditions.

The amplitudes and latencies of the two long-latency components (N100 and LPC) were determined as follows. First, we determined the regions of interest (ROIs) for N100 and LPC from the TEP waveforms and scalp topographies (see **Figures 5 and 6**). Since the distributions of the N100 were lateralized to the stimulated hemisphere, the ROIs of the N100 were defined as nine electrodes around FC1 (F3, F1, Fz, FC3, FC1, FCz, C3, C1, and Cz) in the contralateral TMS condition and nine electrodes around FC2 (Fz, F2, F4, FCz, FC2, FC4, Cz, C2, and C4) in the ipsilateral TMS condition. On the other hand, the ROI of the LPC was defined as nine electrodes around Cz (FC1, FCz, FC2, C1, Cz, C2, CP1, CPz, and CP2) in both TMS side conditions. In the averaged waveforms in the ROI of the N100, the amplitudes and latencies were measured at the largest negative peak during 80–120 ms after TMS. In the averaged waveforms in the ROI of the LPC, amplitudes, and latencies were also measured at the largest positive peak during 120–300 ms after TMS.

### STATISTICAL ANALYSIS

The main purpose of this study was to examine the effects of 2 trial (go or stop) × 2 TMS time (-500 or 0 ms) × 2 TMS side (contralateral or ipsilateral) conditions on the TEPs and MEPs during the performance of a go/stop task. In addition, we needed to confirm the effects of TMS sides on task performance (mean go RT, 50% ST, and SSRT in no-TMS trials). Task performances were compared by two-sample *t*-tests between the contralateral and ipsilateral TMS conditions. Mean MEP amplitudes and the N100 and LPC amplitudes and latencies for the 2 × 2 × 2 conditions were submitted to three-way mixed factorial ANOVAs with within-participant factors of trial and TMS time and the between-participants factor of TMS side. If necessary, *post hoc* multiple comparisons were conducted by using paired *t*-tests with Bonferroni correction. The level of significance that was used for all of the tests was *p* < 0.05.

## RESULTS

### TASK PERFORMANCE

**FIGURE 2:**
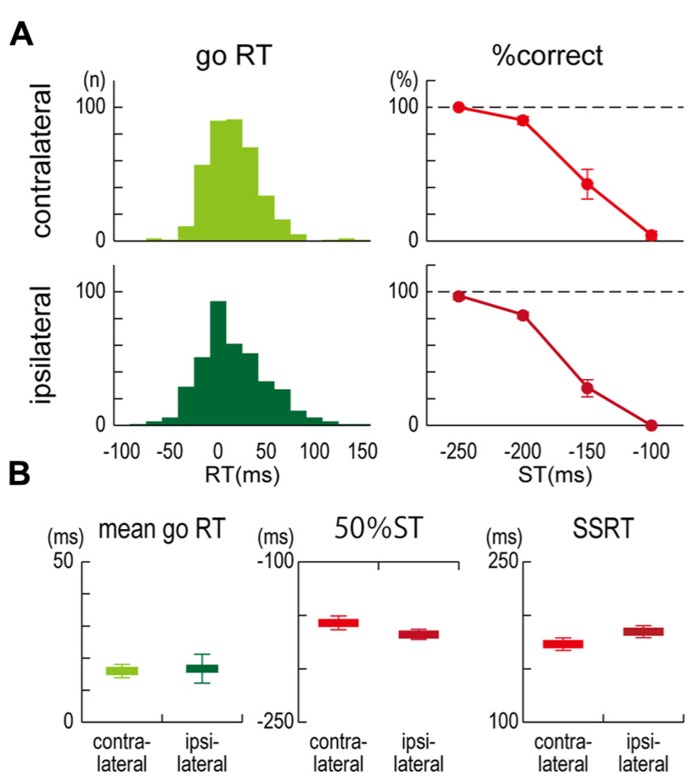
**Task performance.(A)** Distributions of response time (RT) in go trials without TMS and the percentage of correct responses (% correct) in stop trials without TMS for each stop time (ST) that was obtained from six participants in contralateral (upper) and ipsilateral (lower) TMS conditions. RT denotes a clicking time relative to the target. **(B)** Group results of mean go RT (left), estimated 50% STs in stop trials (center), and stop-signal reaction times (SSRTs) in stop trials (right). The 50% ST is an estimated stop time in which the probability of successful stopping was 50%. SSRT is the estimated time required for an unobservable stop process. Error bars show standard error (SE).

In both the contralateral and ipsilateral TMS conditions, all participants could click close to but a little behind the target in the no-TMS go trials, and the longer the time until the target, the more successfully they could stop their clicking in the no-TMS stop trials (**Figure 2A**). For the mean go RTs (contralateral, 16.0 ± 5.1 ms; ipsilateral, 16.7 ± 11.0 ms), 50% STs (contralateral, -157.1 ± 16.0 ms; ipsilateral, -167.9 ± 11.7 ms), and SSRTs (contralateral, 173.0 ± 14.2 ms; ipsilateral, 184.6 ± 13.6 ms) in the no-TMS trials, no significant differences were observed between the contralateral and ipsilateral TMS conditions (**Figure 2B**). These results indicated that task performances in the go/stop task were not very different between the participants in the contralateral and ipsilateral TMS conditions.

### MEP AMPLITUDE

**FIGURE 3:**
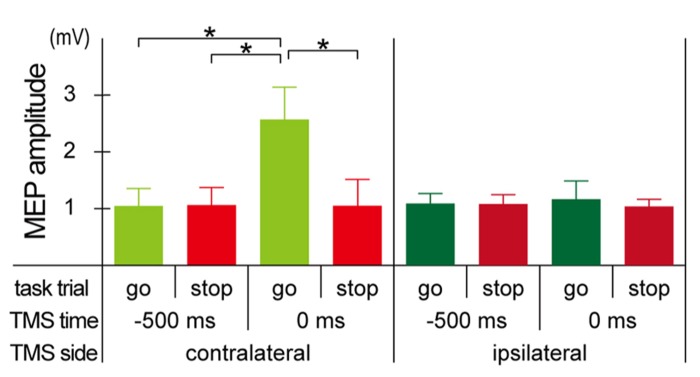
**Motor-evoked potential (MEP) amplitude.** Group means (±SE) of MEP amplitudes during the go/stop task with contralateral (upper) and ipsilateral (lower) TMS at -500 and 0 ms. Error bars show standard error (SE). ^*^p < 0.05; significant difference in *post hoc* multiple comparisons with Bonferroni corrected paired *t*-tests.

Mean MEP amplitudes increased only in the go trials with the contralateral TMS at 0 ms, and there were not much differences in the mean MEP amplitudes in the other seven conditions (**Figure 3**). The mixed factorial ANOVA of the mean MEP amplitudes revealed significant within-participant effects of trial [*F*(1, 10) = 17.7, *p* < 0.01] and TMS time [*F*(1, 10) = 11.4, *p* < 0.01] and their significant interaction [*F*(1, 10) = 17.4, *p* < 0.01]. There were no significant between-participants effects of TMS side, while there were significant TMS side × TMS time [*F*(1, 10) = 10.4, *p* < 0.01], TMS side × trial [*F*(1, 10) = 12.4, *p* < 0.01], and TMS side × TMS time × trial [*F*(1, 10) = 12.8, *p* < 0.01] interactions. Therefore, *post hoc* multiple comparisons were conducted for all six pairs among 2 TMS time × 2 trial conditions separately for each TMS side. There were significant differences of mean MEP amplitudes in three pairs between go trial with TMS at 0 ms and the other three conditions for the contralateral TMS condition, while there was no significant difference of mean MEP amplitudes in all six pairs for the ipsilateral TMS condition. These results indicated that MEP enlarged only in go trials with the contralateral TMS at 0 ms.

### AVERAGED EEGS IN NO-TMS TRIALS

**FIGURE 4:**
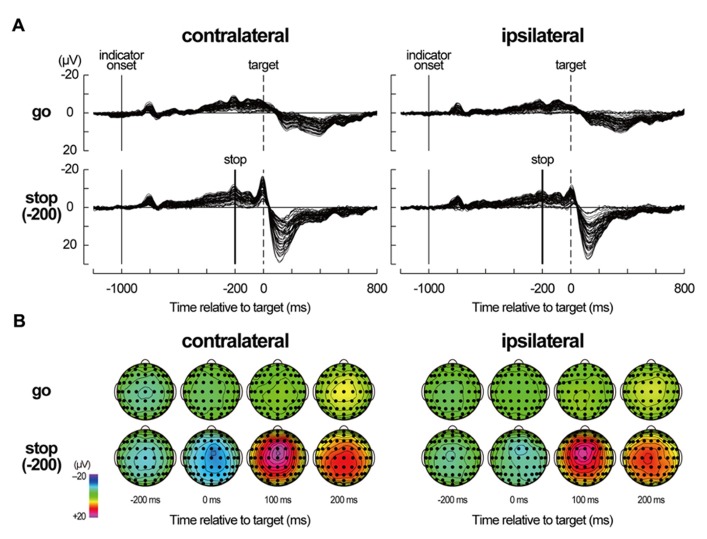
**Averaged electroencephalography (EEG) data in no-TMS trials. (A)** Averaged EEG waveforms in no-TMS trials during a go/stop task for six participants in a contralateral-TMS session (left two panels) and for six participants in an ipsilateral-TMS session (right two panels). For stop trials, only the waveforms in stop trials with a stop time of -200 ms [stop(-200) trials] are displayed. The waveforms of all 61 sites are shown as thin black lines. The vertical thin lines represent indicator onset, the vertical dashed lines represent target time for the go task, and the vertical thick lines represent stop-signal onset. Time scales relative to target are displayed at the bottom. **(B)** Scalp topographies of averaged EEGs in the go and stop(-200) trials for six participants in a contralateral-TMS session (left 2 × 4 arrays) and for six participants in an ipsilateral-TMS session (right 2 × 4 arrays). The topographies are displayed only at -200, 0, 100, and 200 ms relative to target.

In the grand-mean-averaged EEG waveforms in no-TMS trials (**Figure 4**), gradual negative deflections over the fronto-central sites were observed as the target time approached in both go and stop trials. After the stop signal onset of -200 ms, the grand-mean-averaged EEG waveforms clearly differentiated between go and stop trials. Distinct negative–positive peaks over the frontocentral sites appeared around and after the target time in stop trials, while a mild positive peak over the centroparietal sites appeared after the target time in go trials. These waveforms in the no-TMS trials have been typically shown in the go and stop trials of the go/stop (or stop-signal) task (De Jong et al., 1990; Schmajuk et al., 2006).

### TMS-EVOKED POTENTIALS

**FIGURE 5:**
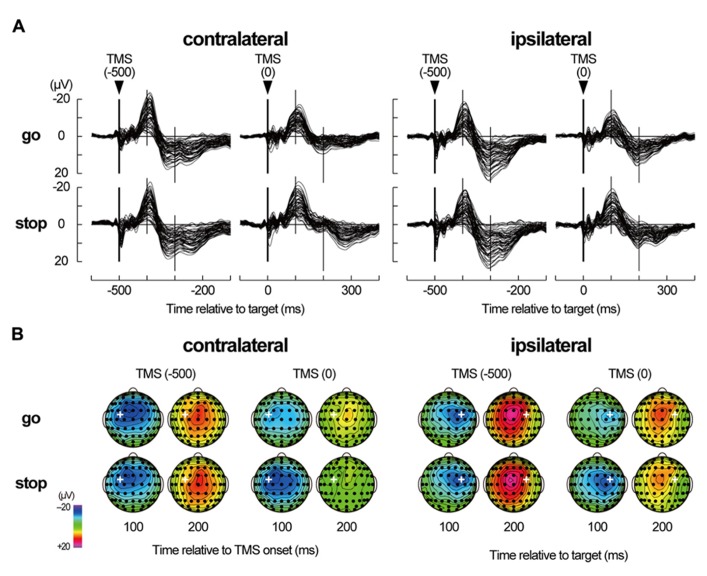
**TMS-evoked potentials. (A)** TMS-evoked potentials (TEPs) during go/stop task with TMS at -500 and 0 ms for six participants in a contralateral-TMS session (left 2 × 2 panels) and with TMS at -500 and 0 ms for six participants in an ipsilateral-TMS session (right 2 × 2 panels). The vertical thick lines represent TMS onset, and the vertical thin lines represent 100 and 200 ms after the TMS onset. Time scales relative to target are displayed at the bottom. (**B)** The scalp topographies of TEPs in the go and stop trials for six participants in a contralateral-TMS session (two left 2 × 4 arrays) and for six participants in an ipsilateral-TMS session (two right 2 × 4 arrays). The bold white plus in each topography represents the TMS sites. The topographies are displayed only at 100 and 200 ms relative to TMS onset, corresponding approximately to N100 and later positive component (LPC), respectively.

In the grand-mean TEPs of all 2 × 2 × 2 conditions (**Figure 5**), a prominent negative peak around the TMS sites was observed about 100 ms after the TMS onset (N100) after the short-latency, high-frequency oscillations. Then, a less pronounced positive deflection over the broad areas that was centered at the midline-central site appeared about 180–300 ms after TMS onset (LPC). Distinct large long-latency negative–positive deflections in TEPs have been typically shown in previous TMS-EEG studies (Paus et al., 2001; Nikulin et al., 2003; Komssi et al., 2004; Bonato et al., 2006; Kičić et al., 2008; Bonnard et al., 2009; Lioumis et al., 2009; Ferreri et al., 2011, 2012; Rogasch et al., 2013).

**FIGURE 6:**
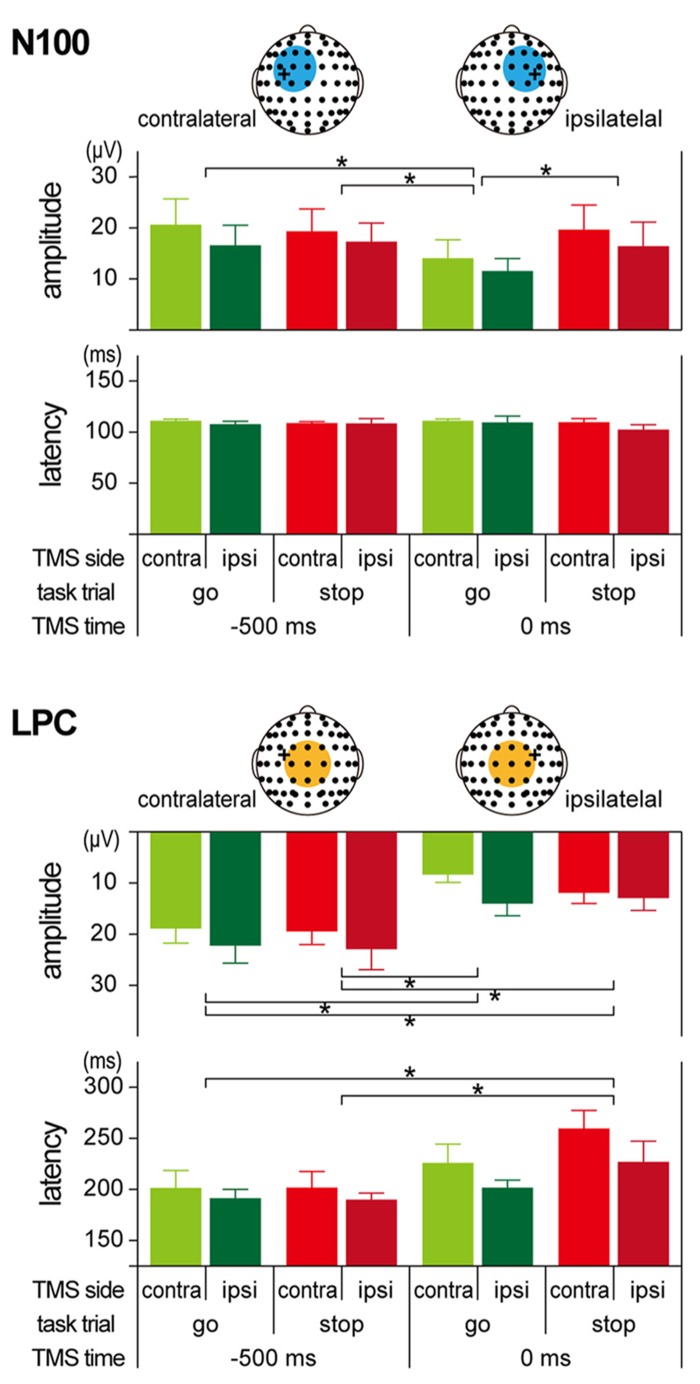
**Amplitudes and latencies of long-latency components in TMS-evoked potential (TEP).** Group means (±SE) of N100 (upper) and LPC (lower) amplitudes and latencies during go/stop tasks with contralateral and ipsilateral TMS at -500 and 0 ms. Light blue and orange circles in the scalp electrode drawings represent regions of interest (ROIs). Error bars show standard error (SE). ^*^p < 0.05; significant difference in *post hoc* multiple comparisons with Bonferroni corrected paired *t*-tests.

Regardless of the TMS sides, N100 amplitudes in go trials with TMS at 0 ms (contralateral, -14.0 ± 8.9 μV; ipsilateral, -11.5 ± 6.1 μV) were smaller than those in go trials with TMS at -500 ms (contralateral, -20.6 ± 12.4 μV; ipsilateral, -16.6 ± 9.7 μV), while there was not much difference in N100 amplitudes in stop trials with TMS at -500 ms (contralateral, -19.3 ± 10.8 μV; ipsilateral, -17.2 ± 9.1 μV) and 0 ms (contralateral, -19.6 ± 11.8 μV; ipsilateral, -16.4 ± 11.7 μV; **Figures 5 and 6**, upper). The mixed factorial ANOVA of N100 amplitudes revealed significant effects of trial [*F*(1, 10) = 12.9, *p* < 0.01] and TMS time [*F*(1, 10) = 18.4, *p* < 0.01] and a significant interaction of them [*F*(1, 10) = 6.2, *p* < 0.05]. However, it showed no significant effects of TMS side and second- and third-interactions, including TMS side. Therefore, *post hoc* multiple comparisons were conducted for all six pairs among 2 TMS time × 2 trial conditions without respect to the TMS side. There were significant differences in the N100 amplitudes in three pairs between go trial with TMS at 0 ms and the other three conditions. In contrast to the N100 amplitudes, there were not much differences in the N100 latencies among the 2 × 2 × 2 conditions (**Figure 6**, upper). The mixed factorial ANOVA of N100 latencies revealed neither significant main effects nor significant interactions. These results indicated that, regardless of the contralateral or ipsilateral TMS, N100 appeared at almost the same latencies, but it was attenuated only in go trials with TMS at 0 ms.

Regardless of the TMS sides, LPC amplitudes in go trials with TMS at 0 ms (contralateral, 8.4 ± 3.8 μV; ipsilateral, 14.0 ± 5.9 μV) were smaller than those in go trials with TMS at -500 ms (contralateral, 18.9 ± 7.1 μV; ipsilateral, 22.2 ± 8.5 μV), and LPC amplitudes in stop trials with TMS at 0 ms (contralateral, 11.9 ± 5.2 μV; ipsilateral, 12.9 ± 5.9 μV) were smaller than those in stop trials with TMS at -500 ms (contralateral, 19.5 ± 6.3 μV; ipsilateral, 22.9 ± 9.8 μV; **Figures 5 and 6**, lower). In addition, LPC latencies in the go and stop trials with TMS at 0 ms were larger than those in go and stop trials with TMS at -500 ms (**Figure 6**, lower). The mixed factorial ANOVA of LPC amplitudes revealed significant effects of TMS time [*F*(1, 10) = 49.9, *p* < 0.01] but no significant effects of trial and no significant interaction of trial × TMS time. Moreover, it also showed no significant effects of TMS side and second- and third-interactions including TMS side. Therefore, *post hoc* multiple comparisons were conducted for all six pairs among 2 TMS time × 2 trial conditions without respect to the TMS side. There were significant differences in the LPC amplitudes in four pairs between both go and stop trial with TMS at -500 ms and both go and stop trial with TMS at 0 ms. The mixed factorial ANOVA of LPC latencies revealed significant effects of TMS time [*F*(1, 10) = 15.9, *p* < 0.01] but no significant effects of trial and no significant interaction of trial × TMS time. Moreover, it also showed no significant effects of TMS side and second- and third-interactions including TMS side. Therefore, *post hoc* multiple comparisons were conducted for all six pairs among 2 TMS time × 2 trial conditions without respect to the TMS side. There were significant differences in the LPC latencies in two pairs between go and stop trial with TMS at -500 ms and stop trial with TMS at 0 ms. These results indicated that, regardless of contralateral or ipsilateral TMS, LPC was reduced and delayed in both go and stop trials with TMS at 0 ms.

## DISCUSSION

In this study, we compared two distinct long-latency components of TEPs (N100 and LPC) at the preparatory, executive, and inhibitory periods during a go/stop task. Consequently, the N100 and LPC of the TEPs were obviously modulated depending on the TMS time and trial conditions. First, the task performance in no-TMS trials were typically shown in the go and stop trials of the go/stop (or stop-signal) task (Logan and Cowan, 1984; Logan et al., 1984; Coxon et al., 2006, 2007; Verbruggen and Logan, 2008). Next, the TEPs were obtained from the subtracted waveforms: the averaged EEG responses in go or stop trials without TMS were subtracted from those in go or stop trials with TMS, respectively. That is, the effects of overlapped event-related potentials during a go/stop task have been excluded from the TEP waveforms, indicating different spatiotemporal patterns of cortical responses to the TMS in a go or stop trial 500 ms before or just at the target time. Therefore, TEP modulations in the task conditions that were demonstrated in this study were considered to be representative of TMS-induced neuronal reactions in the motor cortex and subsequent their propagation to surrounding cortical areas during motor preparation, execution, and inhibition.

Before reaching such a conclusion, some methodological limitations of this study should be discussed. First, the number of participants was small (six for contralateral and ipsilateral condition each). Data from such few participants is easily affected by outliers and therefore we need to interpret them carefully. Second, the long-latency components of the TEP involved not only direct cortical effects of TMS, but also indirect effects that accompanied the TMS, such as auditory and bone-conduction sound by the coil click, somatosensory sensation on the scalp, and afferent proprioceptive/tactile input from twitching muscles (Nikouline et al., 1999; Tiitinen et al., 1999; Nikulin et al., 2003). Some recent studies have used a masking noise for reducing auditory coil-click perception (Tiitinen et al., 1999; Massimini et al., 2005, 2007; Bonnard et al., 2009; Ferreri et al., 2011, 2012; Rogasch et al., 2013). In contrast, we did not take any special precautions for these indirect effects except for the use of earplugs because such techniques will not eliminate all indirect TMS effects from the TEP waveforms completely. Therefore, it cannot be ruled out that the TEP modulations among the task conditions that were demonstrated in this study were influenced by the indirect effects that accompani-ed TMS.

However, considering the within-participant equivalence of the indirect TMS effects that were involved in TEPs, it was unlikely that there was a critical difference in the indirect TMS effects, at least in the within-participant comparisons (2 TMS time × 2 trial conditions) in our experimental settings. Next, previous studies reported that the EEG waveforms that were induced only by coil-click sounds (auditory N1-P2 complex) differed from the TEP waveforms (Nikulin et al., 2003). In this study, apart from approximately symmetric auditory evoked potentials (Mäkinen et al., 2005; Fuentemilla et al., 2006), TEP (especially N100) distributions were asymmetric and lateralized to the stimulated hemisphere (see **Figure 5**), suggesting that non-auditory effect might involve the TEPs. Finally, Paus et al. (2001) and Nikulin et al. (2003) have demonstrated that N100 amplitudes did not correlate with MEP amplitudes in target hand muscles, suggesting that the N100 might not be a predominant reflection of peripheral afferent sensation. In this study, N100 attenuation was accompanied with the MEP amplitude enhancement in the contralateral TMS condition but it was observed without the MEP amplitude enhancement in the ipsilateral TMS conditions. If the N100 attenuation was related to the afferent proprioceptive/tactile input from twitching muscles, it was developed differently in contralateral and ipsilateral TMS conditions. Therefore, it is unlikely that the N100 is a primary reflection of the reafferent proprioceptive/tactile input. When we take all things together, the N100 and LPC modulations among the task conditions seemed to be potential changes that originated from, at least in part, TMS-induced neuronal reactions in the motor cortex and subsequent their propagation to surrounding cortical areas.

The two long-latency TEP components (N100 and LPC) that we focused on corresponded to the last two dominant peaks in typical 300 ms-long waveforms that are induced by TMS to the motor cortex (Bonato et al., 2006; Komssi and Kähkönen, 2006; Lioumis et al., 2009; Ilmoniemi and Kičić, 2010; Ferreri et al., 2011, 2012). N100 has been demonstrated to be very sensitive to small changes in cortical excitability and therefore to be associated with cortical inhibitory process (Nikulin et al., 2003; Bender et al., 2005; Kähkönen and Wilenius, 2007; Kičić et al., 2008). Moreover, recent studies investigating the detailed characteristics of long-interval cortical inhibition induced in the MEPs and TEPs by paired-pulse TMS-EEG paradigms (Fitzgerald et al., 2009; Rogasch et al., 2013) have suggested that the N100 that is evoked by the conditioning TMS is consistent with the underlying mechanism that results in long-interval cortical inhibition of MEPs, which most likely involve GABA_B_-mediated inhibition of cortical activity. However, Ferreri et al. (2011) have recently proposed that the long latency and wide distribution of LPC (P190) suggest the engagement of a reverberant corticosubcortical circuit.

The main finding of this study was the different N100 and LPC modulations between go and stop trials. Although there were not much differences between go and stop trials in the TEP waveforms that were induced by both contralateral and ipsilateral TMS at -500 ms, there were distinct differences between go and stop trials in those at 0 ms. These results indicated that the TEP waveforms were modulated by the underlying cortical and/or subcortical activities that were required for performing the go/stop task. N100 distribution was lateralized to the TMS site, and N100 amplitude was decreased only in go trials with both contralateral and ipsilateral TMS at 0 ms, which was in agreement with the results of previous studies (Nikulin et al., 2003; Kičić et al., 2008;). This decrease of the N100 amplitudes on both sides during go trials might reflect decreased activity in the cortical inhibitory circuit for initiating and executing a planned motor response. Unlike in the case of the contralateral N100, the decrease in the ipsilateral N100 amplitude was not accompanied with an increase in MEP. This inconsistency in TEP and MEP modulations between contralateral and ipsilateral TMS might be due to a methodological difference: TEP is a method that is used for assessing cortical states directly from cortical responses against TMS while the MEP is a method that is used for assessing cortical states indirectly through muscle twitch, including spinal and peripheral effects. However, regardless of the TMS side, the N100 amplitudes in stop trials with TMS at 0 ms were similar in size to those in go and stop trials with TMS at 0 ms. These large N100s in preparatory (at -500 ms in go and stop trials) and inhibitory (at 0 ms in stop trials) periods might reflect enhanced activity in the cortical inhibitory circuit for waiting and inhibiting a planned motor response. As for LPC with TMS at 0 ms, amplitude was decreased and latency was delayed in both go and stop trials. Although LPC has been discussed less frequently than N100 in previous studies (Ferreri et al., 2011), these LPC modulations might also be associated with the state of reverberant corticosubcortical circuits during the performance of a go/stop task.

Recent studies on the short-latency component of TEPs have suggested that they can be used to evaluate reactivity in the stimulated cortex (Bonato et al., 2006; Veniero et al., 2010, 2013; Ferreri et al., 2011). In contrast, we used an IC analysis with extended infomax algorithm (Bell and Sejnowski, 1995; Lee et al., 1999) to identify and boldly remove large TMS-related artifacts and eye-blink- and/or eye-movement-related activities (Jung et al., 2000a,b; Johnson et al., 2012) based on the plausible criteria described above. Although we consequently obtained the TEP waveforms with little distortion during the time period between the appearance of the two large long-latency TEP components, we cannot rule out the possibility that components that were related to not only TMS artifacts, but also the reactivity in the stimulated cortex, were removed particularly during the time period for the TEP components with a shorter latency than N100. Therefore, we discussed only the two large long-latency TEP components and not the TEP components with a shorter latency than N100. Further investigations with EEG data with more careful recordings (Sekiguchi et al., 2011) and stricter TMS artifact-rejection criteria (Veniero et al., 2009) will allow us to understand short-latency cortical reactivity in the motor cortex during motor execution and inhibition.

In summary, we conducted combined TMS-EEG recordings during a go/stop task and compared the two dominant long-latency components of the TEPs (N100 and LPC) at the preparatory, executive, and inhibitory periods during go/stop tasks. Consequently, N100 and LPC were differently modulated in go and stop trials that were conducted with contralateral and ipsilateral TMS just at the target time. The N100 amplitude was decreased only in go trials while the LPC amplitude was decreased and the LPC latency was delayed in both go and stop trials. These results suggested that TMS-induced neuronal reactions in the motor cortex and subsequent their propagation to surrounding cortical areas might change functionally according to task demand in go and stop trials, that is, motor preparation, execution, and inhibition.

## Conflict of Interest Statement

The authors declare that the research was conducted in the absence of any commercial or financial relationships that could be construed as a potential conflict of interest.
